# Higher fibre intake is associated with lower blood pressure levels in patients with type 1 diabetes

**DOI:** 10.1186/1758-5996-7-S1-A6

**Published:** 2015-11-11

**Authors:** Mileni Vanti Beretta, Fernanda R Bermaud, Ciglea do Nascimento Schaan, Thais Steemburgo, Ticiana da Costa Rodrigues

**Affiliations:** 1 UFRGS, Porto Alegre, Brazil

## Background

The present investigation sought to evaluate the potential association between dietary fiber intake and blood pressure in adult patients with type 1 diabetes (T1D). A cross-sectional study was carried out in 111 outpatients with T1D from Porto Alegre, Brazil. Patients were predominantly male (56%) and white (88%), with a mean age of 40±10 years, diabetes duration of 18±9 years, body mass index (BMI) 24.8±3.85 kg/m2, and HbA1c 9.0±2.0%. After clinical and laboratory evaluation, patients completed 3-day weighed food records. Adequacy was confirmed by estimation of protein intake from urinary urea nitrogen. Patients were stratified into two groups according to adequacy of fibre intake in relation to American Diabetes Association recommendations: below recommended daily intake (<14g fibre/1000 kcal) or at/above recommended intake (≥14g/1000 kcal). Patients in the higher fibre intake group exhibited significantly lower systolic blood pressure (SBP) (115.9±12.2 vs 125.1±25.0 mmHg, p=0.016) and diastolic blood pressure (DBP) (72.9±9.2 vs 78.5±9.3 mmHg, p=0.009), higher energy intake (2164.0±626.0 vs 1632.8±502.0 kcal, p<0.001), and lower BMI (24.4±3.5 vs 26.2±4.8, p=0.044). Linear regression modelling, adjusted for age, energy intake, sodium intake, and BMI, indicated that higher fiber intake was associated with lower SBP and DBP. No significant between-group differences were observed with regard to duration of diabetes, glycaemic control, insulin dosage, presence of nephropathy, or retinopathy. We conclude that fibre consumption meeting or exceeding current adequate intake recommendations is associated with lower SBP and DBP in patients with T1D.

